# Stable HIV-1 integrase diversity during initial HIV-1 RNA Decay suggests complete blockade of plasma HIV-1 replication by effective raltegravir-containing salvage therapy

**DOI:** 10.1186/1743-422X-10-350

**Published:** 2013-12-05

**Authors:** Marc Noguera-Julian, Maria Casadellà, Christian Pou, Cristina Rodríguez, Susana Pérez-Álvarez, Jordi Puig, Bonaventura Clotet, Roger Paredes

**Affiliations:** 1 IrsiCaixa AIDS Research Institute-HIVACAT, Hospital Universitari Germans Trias I Pujol, Ctra de Canyet s/n, Badalona 08916, Catalonia, Spain; 2 HIV Unit&Fundació Lluita contra la SIDA Hospital Universitari Germans Trias i Pujol, Badalona, Catalonia, Spain; 3 Universitat Autònoma de Barcelona, Badalona, Catalonia, Spain

**Keywords:** HIV-1, Raltegravir, Dynamics, Deep sequencing

## Abstract

**Background:**

There is legitimate concern that minority drug-resistant mutants may be selected during the initial HIV-1 RNA decay phase following antiretroviral therapy initiation, thus undermining efficacy of treatment. The goal of this study was to characterize viral resistance emergence and address viral population evolution during the first phase of viral decay after treatment containing initiation.

**Findings:**

454 sequencing was used to characterize viral genetic diversity and polymorphism composition of the HIV-1 integrase gene during the first two weeks following initiation of raltegravir-containing HAART in four ART-experienced subjects. No low-prevalence Raltegravir (RAL) drug resistance mutations (DRM) were found at baseline. All patients undergoing treatment received a fully active ART according to GSS values (GSS ≥ 3.5). No emergence of DRM after treatment initiation was detected. Longitudinal analysis showed no evidence of any other polymorphic mutation emergence or variation in viral diversity indexes.

**Conclusions:**

This suggests that fully active salvage antiretroviral therapy including raltegravir achieves a complete blockade of HIV-1 replication in plasma. It is unlikely that raltegravir-resistant HIV-1 may be selected in plasma during the early HIV-1 RNA decay after treatment initiation if the administered therapy is active enough.

## Findings

There is legitimate concern that minority drug-resistant mutants may be selected during the initial HIV-1 RNA decay phase following antiretroviral therapy initiation, when decreasing but still detectable HIV-1 RNA levels transiently coexist with suboptimal drug levels. Studies using allele-specific PCR suggested that drug-resistant HIV-1 might be selected and replicate in the first weeks of suppressive antiretroviral therapy (ART) [1,2]. If they were confirmed, such observations would support using intensified ART regimens to accelerate viremia suppression and curtail drug resistance evolution, but not only in treatment-naïve subjects initiating first-line ART. Achieving fast blockade of HIV replication would even be more important in subjects with drug-resistant HIV-1 initiating salvage ART, given that they are more vulnerable to drug resistance evolution and are closer to exhausting their treatment options.

In the past, however, first-line treatment with 4, 5, or more antiretrovirals did not produce better virological outcomes than standard 3-drug ART [3]. Indeed, evolutionary studies in macaque models [4] suggested that viral replication was completely shut down by therapy and that development of antiretroviral drug resistance during the initial viremia decay was unlikely to occur, provided that treatments were active enough and resistant variants did not pre-exist. If HIV replication could be shut down with standard ART, addition of further drugs to ART regimens would not provide additional protection against drug resistance.

Mathematical models of HIV resistance kinetics predict faster selection of resistant viruses with more potent regimens. On the other hand, previous studies correlated higher resistance selection with longer time to achieving undetectable HIV-1 RNA levels [1,2]. raltegravir (RAL) achieves fast viremia suppression when combined with an optimized backbone treatment (OBT) [5], but has a low-genetic barrier and HIV-1 variants with single raltegravir drug resistance mutations could pre-exist at low levels [6,7].

We thus sought to explore in a proof-of-concept study whether raltegravir-resistant mutants were selected during the initial HIV-1 RNA decay following initiation of raltegravir-containing ART in 4 treatment-experienced subjects with HIV-1 resistance to at least 2 antiretroviral drug classes using ultrasensitive, quantitative 454 sequencing. We also sought to characterize the diversity and viral population shifts in HIV-1 integrase during this initial HIV-RNA decay phase.

### Study design

The study included 4 ART-experienced HIV-1-infected subjects who initiated salvage ART including raltegravir (RAL) with more than 3 active drugs (Table 1). All subjects provided signed informed consent to participate into the study. The Ethics Committee of the “Germans Trias i Pujol” Hospital, approved the study on 21 December 2007, reference #: AC-07-107. Integrase sequencing was performed using 454 Genome Sequencer FLX (Life Sequencing/Roche) in consecutive plasma samples with detectable viral loads on days 0 to 14 following RAL initiation. 454 sequencing of integrase was performed using a four-amplicon design for robustness (see Additional file 1). A homology filter was applied to rule out pNL43 contamination. AVA v2.8 software was used to obtain mutation prevalence and unique consensus sequences, which were further processed. Short consensus sequences (less than 90% of the expected length) were filtered out. Aminoacid variant calling was realized using in-house perl code on pairwise alignments over HXB2R reference sequence, discarding those sequences with in-frame STOP codons. In order to discard strand-dependent sequencing errors, only variants which were present both in the forward and reverse strand and presented a forward/reverse prevalence frequency ratio within 1 log were accepted. GSS scores were calculated using Stanford HIVdb guidelines. MAFFT (v6.815b) software [8] was then used to perform multiple alignment on consensus sequences. Shannon entropy was calculated for each amplicon using the alignment obtained and the prevalence information kept for each position. Mean pairwise distances (both intra amplicon and relative to an HXB2 reference) were calculated using a Kimura-80 model and sequence prevalence information with *R/ape* package [9].

**Table 1:** Subject clinical details

**Subject**	**DS1**	**DS2**	**DS3**	**DS4**
**Gender**	Male	Male	Male	Female
**Age**	61	39	49	44
**Date of diagnostic**	13/05/96	01/01/90	01/07/87	01/01/91
**Transmission type**	Heterosexual	IVDU	IVDU	IVDU
**Previous ART lines**	9	6	12	12
**#drugs before RAL**	13	9	13	12
**New drugs started alongside RAL**	DRV	DRV	DRV,ETV, T-20	DRV,MVC
**Previous exposure**				
**NRTI**	YES	YES	YES	YES
**NNRTI**	YES	YES	YES	YES
**PI**	YES	YES	YES	YES
**T-20**	NO	NO	NO	NO
**ART**	ddI + 3TC + DRV/r + RAL	TDF/FTC + DRV/r + RAL	TDF/FTC + DRV/r + RAL + T-20	DRV/r + RAL + MVC
**GSS**	4	3.5	3.5	3.5
**Baseline CD4 (cells/mm**3**)**	614	300	332	399

Intravenous Drug Use (IVDU); Genotypic Sensitivity Score (GSS, Stanford HIVdb), NRTI: Nucleoside-analogues Reverse Transcriptase Inhibitor; NNRTI non- Nucleoside-analogues Reverse Transcriptase Inhibitor; PI: Protease Inhibitor; Darunavir (DRV); Didanosine (DDI); Lamivudine (3TC); Raltregravir (RAL); Ritonavir (RTV); Emtricitabine (FTC); Tenofovir (TDF); Etravirine (ETV); Enfuvirtide (T-20); Maraviroc (MVC).

## Results

The genotypic susceptibility score (GSS) of the salvage treatment was ≥3.5 in all patients according to population sequencing. Sensitivity scores were further confirmed considering 454 deep sequencing data obtained for integrase and protease/retrotranscriptase, when available (see Additional file 2: Table S1). At baseline, median (interquartile range) for HIV-1 ARN and CD4+ T cell counts were, 116.500 (57.750; 215.000) copies/mL and 399 (300;614) cells/mm3. Viral load decline at day 7, when available, was more than 1log copies/mL.

**Figure 1:**
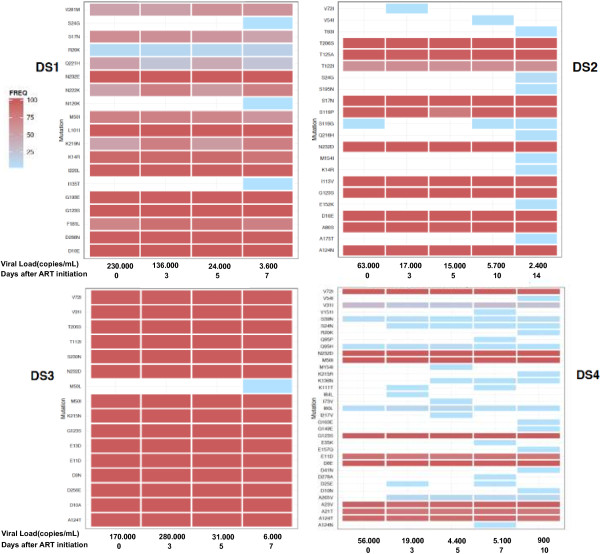
**Longitudinal evolution of integrase polymorphisms relative to Consensus B.** Each plot corresponds to one subject. Integrase polymorphisms are in the vertical axis. The horizontal axis shows the different longitudinal timepoints assessed during the initial HIV-1 RNA load decay. The frequency of each polymorphism is represented using a color scale, from 100% (dark red) to a 1.0% threshold (light blue). White indicates non detection of polymorphisms. A remarkable stability in polymorphism type and frequency is observed. Of note, none of the IAS-list (2013) integrase resistant mutations were found.

The median number of sequences obtained from 454 sequencing per amplicon was 3404 (IQR: 1497–5304). Initiation of treatment with raltegravir was associated with a rapid decline in HIV-1 RNA levels but no changes in integrase diversity or shifts relative to an HXB2R external reference (see Additional file 3: Figures S1, S2 and Additional file 2: Table S2). The frequency of major integrase polymorphisms remained stable during the viral load decay phase (Figure 1). In addition, longitudinal 454 sequencing of integrase gene did not result in the emergence of resistant mutations or any of the detected polymorphisms and, so, sensitivity to RAL remained unchanged during the first two weeks and none of the minor viral populations carrying minor polymorphisms were selected.

## Discussion

We observed stability of HIV-1 integrase diversity and heterogeneity within the first phase of HIV-1 RNA decay. The type and frequency of polymorphisms in sequential samples during early HIV-1 RNA decay suggests that salvage antiretroviral therapy including raltegravir is able to achieve an effective blockade of HIV-1 replication in plasma, even in heavily treatment-experienced individuals, in a context where a potent antiretroviral regimen, defined by a GSS ≥ 3, can be constructed. Of note, the rapid decline on HIV-1 RNA levels may also affect the capability of detecting minor polymorphisms using 454. When the number of obtained sequences is higher than the number of expected initial viral templates, re-sequencing of the same minor variants may lead to an artifactual appearance of low prevalence polymorphisms. Interestingly, minority polymorphisms were more frequent and fluctuated at later time points, at very low levels. Thus, obtained minor variant frequencies must be interpreted accounting for sampling limitations. Conversely, sampling limitations are not expected to significantly affect viral diversity due to the much larger impact of highly prevalent variants over minority ones in these measures.

Importantly, the blockade in replication observed in this study must be understood as a result of the global antiretroviral treatment pressure against the whole viral quasi-species. Indeed, additional 454 data on protease and reverse transcriptase at baseline confirms the full potency of the prescribed regimen, as GSS scores were not modified by the few minor resistant variants additionally detected. Our findings contrast with the rapid emergence of raltegravir resistance when non- fully active regimens (GSS < 3) are used [6,7]. Also, antiretroviral drug resistance might evolve in other compartments or reservoirs with low drug penetration in the presence of residual HIV-1 replication. It is also worth noting that the sample size in this study is low (n = 4), thus reducing our ability to observe emergence of resistance, and that GSS in our study ranged from 3.5 to 4, so we cannot conclude whether a GSS = 3 would achieve similar results.

In conclusion, raltegravir-resistant HIV-1 may not be selected in plasma during the early HIV-1 RNA decay after salvage treatment initiation, provided that subjects are given a fully effective regimen that is able to shut down plasma replication. Further studies are required to assess whether similar findings might also be observed with other drugs or regimens and to clarify what is the minimum GSS (i.e. 3, 4 or higher) required to block HIV-1 evolution during HIV-1 RNA decay following ART initiation.

## Competing interests

The authors declared that they have no competing interest.

## Authors’ contributions

RP, BC and MN designed the study. CR, SP, and MN performed the analysis, MC and CP performed 454 sequencing experiments, JP collected all samples. RP and MN drafted the article, which was reviewed, edited and approved by all authors.

## Supplementary Material

Additional file 1 Methods for UDS-454 DNA library preparation and primer design. Click here for file

Additional file 2: Table S1 Drug resistant mutations at baseline for all patients obtained from 454 Data. NRTI: Nucleoside-analogues Reverse Transcriptase Inhibitor; NNRTI non- Nucleoside-analogues Reverse Transcriptase Inhibitor; PI: Protease Inhibitor; INSTI: integrase strand transfer inhibitor. **Table S2.** The evolution of HIV integrase diversity during the initial HIV-1 RNA decay. VL: Viral Load, GSS: HIVdb Genotypic Susceptibility Score, NA: Not available. Mean pairwise distance calculated vs HXB2R. Shannon Entropy Score calculated for haplotype set multiple alignments. **Table S3.** Number of sequence readouts obtained for each Sample/timepoint and Amplicon before and after applying the pNL4-3 contamination filter to raw sequence data. Percent values are shown when ≥0.1%. **Table S4.** Polymorphisms frequencies at baseline as obtained for each amplicon separately. (NC: Not Covered; N/A: No sequence data available). Click here for file

Additional file 3: Figure S1 Longitudinal evolution mean pairwise distance versus an external reference (HXB2R). Each boxplot shows results from HXB2 referenced pairwise distance for the four integrase amplicons. R/ape package, with a Kimura-80 model was used to calculate pairwise distances. **Figure S2.** Longitudinal evolution of summed Shannon Entropy values. Each boxplot shows results from Shannon Entropy values calculated and collapsed for the four integrase amplicons in a particular sample/timepoint combination. Click here for file
