# Supplementary material for: Stable HIV-1 integrase diversity during initial HIV-1 RNA Decay suggests complete blockade of plasma HIV-1 replication by effective raltegravir-containing salvage therapy
Source: Virol J. 2013 Dec 5;10:350. doi: 10.1186/1743-422X-10-350 (PMC3867623; doi:10.1186/1743-422X-10-350)
Supplement: Additional file 1 — Methods for UDS-454 DNA library preparation and primer design. [file 1743-422X-10-350-S1.docx]

**Supplementary Information**

**S1. 454 sequencing**

Integrase gene was reverse transcribed from RNA to DNA and subsequently amplified using primer forward 5’-GGAATTGGAGGAAATGAACAAG-3’ (4170-4191, HXB2R) and reverse 5’-GTACACATCCCACTAGGG-3’ (5203-5220, HXB2R) by one-step RT-PCR using SuperScript III and PlatinumTaq High Fidelity (Life Technologies, Paisley, UK) and the. PCR conditions were 30 min. at 52ºC for retrotranscription step, first denaturalization step of 2 min. at 94ºC, 20 cycles of 2 min. at 94ºC, 30 sec. at 50ºC and 1 min. and 30 sec. at 68ºC, and a final polymerisation step of 5 min. at 68ºC.

Library of amplicons were generated in triplicate from one-step RT-PCR product and then pooled. Nested PCR conditions were a first denaturalization step of 2 min. at 94ºC, 20 cycles of 2 min. at 94ºC, 30 sec. at 50ºC and 45 sec. at 68ºC, and a final polymerisation step of 3 min. at 68ºC using PlatinumTaq High Fidelity (Life Technologies, Paisley, UK). 454 Fusion primers incorporated adaptors A and B, and also the identifiers required for parallel sample sequencing. A set of 4 overlapped amplicons shown in the table below were designed to cover the whole integrase.

| Amplicon # | Direction | Position (HXB2R) | Sequence 5’ - 3’ |
| --- | --- | --- | --- |
| 1 | Forward | 4208-4227 | TGCTGGAATCAGGAAAGTAC |
|  | Rerverse | 4630-4648 | GGATCAAGCAGGAATTTGG |
| 2 | Forward | 4467-4486 | GCCAGTGGATATATAGAAGC |
|  | Reverse | 4786-4804 | AAAGAAAAGGGGGGATTGG |
| 3 | Forward | 4609-4624 | AGGCCGCCTGTTGGTG |
|  | Reverse | 4941-4958 | CCAGCAAAGCTTCTCTGG |
| 4 | Forward | 4758-4778 | CAAATGGCAGTATTCATCCAC |
|  | Reverse | 5102-5126 | GGAAAAGTTTAGTAAAACACCATAT |

Nested PCR products were purified using AMPure Magnetic Beads (Beckman Coulter, Inc, Brea,CA). Concentration and quality of purified PCR products were inspected using fluorometry (PicoGreen, Life Technologies, Paisley, UK) and spectrophotometry (Lab on a Chip, Agilent Technologies, Foster City, CA), respectively. Nested PCR products were generated in triplicate for each amplicon and then pooled into amplicon pools. Next, equimolar amplicon pools were merged to perform emPCR as in. ([10]), adding a ratio 1:1 between molecules and beads. Genome Sequencher FLX (Life Sequencing/Roche) was the platform employed for DNA sequencing.
