# Supplementary material for: Stable HIV-1 integrase diversity during initial HIV-1 RNA Decay suggests complete blockade of plasma HIV-1 replication by effective raltegravir-containing salvage therapy
Source: Virol J. 2013 Dec 5;10:350. doi: 10.1186/1743-422X-10-350 (PMC3867623; doi:10.1186/1743-422X-10-350)
Supplement: Additional file 2: Table S1 — Drug resistant mutations at baseline for all patients obtained from 454 Data. NRTI: Nucleoside-analogues Reverse Transcriptase Inhibitor; NNRTI non- Nucleoside-analogues Reverse Transcriptase Inhibitor; PI: Protease Inhibitor; INSTI: integrase strand transfer inhibitor. Table S2. The evolution of HIV integrase diversity during the initial HIV-1 RNA decay. VL: Viral Load, GSS: HIVdb Genotypic Susceptibility Score, NA: Not available. Mean pairwise distance calculated vs HXB2R. Shannon Entropy Score calculated for haplotype set multiple alignments. Table S3. Number of sequence readouts obtained for each Sample/timepoint and Amplicon before and after applying the pNL4-3 contamination filter to raw sequence data. Percent values are shown when ≥0.1%. Table S4. Polymorphisms frequencies at baseline as obtained for each amplicon separately. (NC: Not Covered; N/A: No sequence data available). [file 1743-422X-10-350-S2.docx]

| **Table S1.** Drug resistant mutations at baseline for all patients obtained from 454 Data. NRTI: Nucleoside-analogues Reverse Transcriptase Inhibitor; NNRTI non- Nucleoside-analogues Reverse Transcriptase Inhibitor; PI: Protease Inhibitor; INSTI: integrase strand transfer inhibitor |
| --- |
| \| Patient ID \| NRTI DRM(%) \| nNRTI DRM(%) \| PI DRM(%) \| INSTI DRM(%) \| \| --- \| --- \| --- \| --- \| --- \| \| DS1 \| K70R(65.1)  D67N(62.4) \| G190A(59.5)  K103N(30.8) \| **L63P**(6.8)  M36I(67.4)  V77I(17.1)  V82A(67.5)  I84V(67.6)  K20R(67.8) \| None found \| \| DS2 \| None found \| E138A(100) \| L63P(99.9)  V77I(99.7)  I93L(99.7) \| **V72I**(0.8)  T206S(100) \| \| DS3^a^ \| M41L, L74V, M184V, T215Y \| K101E, V179FV, G190V \| 32I, M46I, I54V, I84V, L10F, L33I, Q58E, A71V, G73S, L89V \| S230N(100.0) V72I (96.3) **M154I** (0.6) T206S (99.9) \| \| DS4 \| **K219R**(9.7)  **K70R**(7.2) \| **V90I**(6.8)  **A98G**(6.0)  **V179I**(1.0) \| L63P(88.3)  **L90M**(10.5)  I93L(21.4) \| V72I(92.7) \| |
| **^a^**RT/PI inhibitor resistant mutations obtained from Population Sequencing data |

| **Table S2.**The evolution of HIV integrase diversity during the initial HIV-1 RNA decay. VL: Viral Load, GSS: HIVdb Genotypic Susceptibility Score, NA: Not available. Mean pairwise distance calculated vs HXB2R. Shannon Entropy Score calculated for haplotype set multiple alignments. |
| --- |
| \| **PatientID**  **(GSS)** \| **DAY** \| **VL (copies/mL)** \| **# reads** \| **# Unique**  **reads** \| **Mean**  **Pairwise Distance** \| **Global Shannon Entropy** \| \| --- \| --- \| --- \| --- \| --- \| --- \| --- \| \| **DS1 (4)** \| 0 \| 230000 \| 9882 \| 164 \| 0.033 \| 0.014 \| \|  \| 3 \| 136000 \| 16855 \| 225 \| 0.033 \| 0.015 \| \|  \| 5 \| 24000 \| 9850 \| 223 \| 0.039 \| 0.014 \| \|  \| 7 \| 3600 \| 6196 \| 253 \| 0.037 \| 0.018 \| \| **DS2 (3.5)** \| 0 \| 63000 \| 11595 \| 626 \| 0.045 \| 0.022 \| \|  \| 3 \| 17000 \| 26824 \| 639 \| 0.045 \| 0.018 \| \|  \| 5 \| 15000 \| 3983 \| 496 \| 0.045 \| 0.019 \| \|  \| 10 \| 5700 \| 32954 \| 636 \| 0.046 \| 0.018 \| \|  \| 14 \| 2400 \| 39566 \| 451 \| 0.045 \| 0.021 \| \| **DS3 (3.5)** \| 0 \| 170000 \| 9787 \| 120 \| 0.037 \| 0.002 \| \|  \| 3 \| 280000 \| 17612 \| 133 \| 0.035 \| 0.002 \| \|  \| 5 \| 31000 \| 18913 \| 154 \| 0.038 \| 0.002 \| \|  \| 7 \| 6000 \| 3511 \| 127 \| 0.038 \| 0.002 \| \| **DS4 (3.5)** \| 0 \| 56000 \| 3388 \| 319 \| 0.028 \| 0.022 \| \|  \| 3 \| 19000 \| 111022 \| 575 \| 0.034 \| 0.018 \| \|  \| 5 \| 4400 \| 6366 \| 387 \| 0.034 \| 0.019 \| \|  \| 7 \| 5100 \| 91617 \| 567 \| 0.034 \| 0.020 \| \|  \| 10 \| 900 \| 16581 \| 332 \| 0.035 \| 0.022 \| \| **pNL4.3** \| NA \| NA \| 11993 \| 14 \| 0.031 \| 0.000 \| |

**Table S3.** Number of sequence readouts obtained for each Sample/timepoint and Amplicon before and after applying the pNL4-3 contamination filter to raw sequence data. Percent values are shown when ≥0.1%

|  |  | Amp_1 | | Amp_2 | | Amp_3 | | Amp_4 | | Contaminating Sequences (NL4-3) [%] | | | |
| --- | --- | --- | --- | --- | --- | --- | --- | --- | --- | --- | --- | --- | --- |
|  |  | Before | After | Before | After | Before | After | Before | After | Amp_8 | Amp_9 | Amp_10 | Amp_11 |
| DS1 | t0 | 2416 | 2416 | 3267 | 3266 | 0 | 0 | 4199 | 4199 | 0 | 1 | 0 | 0 |
|  | t3 | 2690 | 2690 | 3898 | 3894 | 5978 | 5978 | 4289 | 4289 | 0 | 4 | 0 | 0 |
|  | t5 | 731 | 731 | 1356 | 1356 | 2471 | 2471 | 5292 | 5292 | 0 | 0 | 0 | 0 |
|  | t7 | 2178 | 2177 | 1668 | 1668 | 1559 | 1559 | 791 | 791 | 1 | 0 | 0 | 0 |
| DS2 | t0 | 3404 | 3403 | 4499 | 4495 | 1807 | 1807 | 1885 | 1885 | 1 | 4 | 0 | 0 |
|  | t3 | 4237 | 4236 | 9013 | 9007 | 7039 | 7038 | 6535 | 6534 | 1 | 6[0.1] | 1 | 1 |
|  | t5 | 739 | 739 | 966 | 965 | 1257 | 1257 | 1021 | 1021 | 0 | 1 | 0 | 0 |
|  | t10 | 4862 | 4862 | 6849 | 6846 | 7866 | 7866 | 13377 | 13376 | 0 | 3 | 0 | 1 |
|  | t14 | 1011 | 1011 | 6949 | 6948 | 25371 | 25371 | 6235 | 6235 | 0 | 1 | 0 | 0 |
| DS3 | t0 | 1868 | 1868 | 3452 | 3449 | 2411 | 2411 | 2056 | 2056 | 0 | 3 | 0 | 0 |
|  | t3 | 3059 | 3059 | 4344 | 4343 | 4840 | 4840 | 5369 | 5369 | 0 | 1 | 0 | 0 |
|  | t5 | 3919 | 3919 | 5316 | 5315 | 4818 | 4818 | 4860 | 4860 | 0 | 1 | 0 | 0 |
|  | t7 | 695 | 695 | 918 | 918 | 298 | 298 | 1600 | 1600 | 0 | 0 | 0 | 0 |
| DS4 | t0 | 521 | 521 | 925 | 925 | 1207 | 1197 | 735 | 735 | 0 | 0 | 10 | 0 |
|  | t3 | 15732 | 15731 | 76059 | 76039 | 18411 | 18312 | 820 | 820 | 1 | 20 | 99[0.5] | 0 |
|  | t5 | 1104 | 1104 | 1507 | 1507 | 2268 | 2257 | 1487 | 1487 | 0 | 0 | 11[0.5] | 0 |
|  | t7 | 60621 | 60620 | 13623 | 13621 | 3417 | 3417 | 13956 | 13955 | 1 | 2 | 0 | 1 |
|  | t10 | 2824 | 2824 | 4378 | 4377 | 4391 | 4297 | 4988 | 4988 | 0 | 1 | 94[2.1] | 0 |

**Table S4.** Polymorphisms frequencies at baseline as obtained for each amplicon separately. (NC: Not Covered ; N/A: No sequence data available)

| **Sample** | **Time(days)** |  | **Amp_1** | **Amp_2** | **Amp_3** | **Amp_4** |
| --- | --- | --- | --- | --- | --- | --- |
| **DS1** | **0** | **D10E** | 100.0 | NC | N/A | NC |
|  |  | **K14R** | 91.6 | NC | N/A | NC |
|  |  | **S17N** | 64.8 | NC | N/A | NC |
|  |  | **R20K** | 6.8 | NC | N/A | NC |
|  |  | **M50I** | 73.5 | NC | N/A | NC |
|  |  | **L101I** | 99.9 | 99.9 | N/A | NC |
|  |  | **G123S** | 99.9 | 100.0 | N/A | NC |
|  |  | **F181L** | NC | 71.2 | N/A | NC |
|  |  | **G193E** | NC | NC | N/A | 98.1 |
|  |  | **K219N** | NC | NC | N/A | 48.6 |
|  |  | **I220L** | NC | NC | N/A | 97.5 |
|  |  | **Q221H** | NC | NC | N/A | 50.9 |
|  |  | **N222K** | NC | NC | N/A | 48.7 |
|  |  | **N232E** | NC | NC | N/A | 100.0 |
|  |  | **V281M** | NC | NC | N/A | 49.2 |
|  |  | **D288N** | NC | NC | N/A | 98.7 |
|  |  |  |  |  |  |  |
| **DS2** | **0** | **D10E** | 100.0 | NC | NC | NC |
|  |  | **S17N** | 99.9 | NC | NC | NC |
|  |  | **A80S** | 100.0 | NC | NC | NC |
|  |  | **I113V** | 99.8 | 99.8 | NC | NC |
|  |  | **S119P** | 97.1 | 98.7 | NC | NC |
|  |  | **S119G** | 2.4 | 1.3 | NC | NC |
|  |  | **T122I** | 55.6 | 66.7 | NC | NC |
|  |  | **G123S** | 100.0 | 100.0 | NC | NC |
|  |  | **A124N** | 100.0 | 100.0 | NC | NC |
|  |  | **T125A** | 100.0 | 100.0 | NC | NC |
|  |  | **T206S** | NC | NC | 100.0 | 100.0 |
|  |  | **N232D** | NC | NC | 99.8 | 100.0 |
|  |  |  |  |  |  |  |
| **DS3** | **0** | **D3N** | 100.0 | NC | NC | NC |
|  |  | **D10A** | 99.7 | NC | NC | NC |
|  |  | **E11D** | 100.0 | NC | NC | NC |
|  |  | **E13D** | 100.0 | NC | NC | NC |
|  |  | **V31I** | 100.0 | NC | NC | NC |
|  |  | **M50I** | 99.2 | NC | NC | NC |
|  |  | **V72I** | 97.8 | NC | NC | NC |
|  |  | **T112I** | 99.8 | 100.0 | NC | NC |
|  |  | **G123S** | 100.0 | 100.0 | NC | NC |
|  |  | **A124T** | 99.1 | 99.1 | NC | NC |
|  |  | **T206S** | NC | NC | 100.0 | 99.8 |
|  |  | **K215N** | NC | NC | 99.5 | 99.6 |
|  |  | **S230N** | NC | NC | 100.0 | 100.0 |
|  |  | **N232D** | NC | NC | 100.0 | 100.0 |
|  |  | **D256E** | NC | NC | NC | 100.0 |
|  |  |  |  |  |  |  |
| **DS4** | **0** | **D6E** | 99.4 | NC | NC | NC |
|  |  | **E11D** | 86.4 | NC | NC | NC |
|  |  | **A21T** | 87.5 | NC | NC | NC |
|  |  | **A23V** | 95.8 | NC | NC | NC |
|  |  | **V31I** | 31.1 | NC | NC | NC |
|  |  | **S39N** | 5.0 | NC | NC | NC |
|  |  | **M50I** | 100.0 | NC | NC | NC |
|  |  | **I60L** | 3.8 | NC | NC | NC |
|  |  | **V72I** | 99.6 | NC | NC | NC |
|  |  | **Q95H** | 2.9 | 3.35 | NC | NC |
|  |  | **G123S** | 99.2 | 98.9 | NC | NC |
|  |  | **A124T** | 92.3 | 90.6 | NC | NC |
|  |  | **N232D** | NC | NC | 100.0 | 100.0 |
