# Supplementary material for: Stable HIV-1 integrase diversity during initial HIV-1 RNA Decay suggests complete blockade of plasma HIV-1 replication by effective raltegravir-containing salvage therapy
Source: Virol J. 2013 Dec 5;10:350. doi: 10.1186/1743-422X-10-350 (PMC3867623; doi:10.1186/1743-422X-10-350)
Supplement: Additional file 3: Figure S1 — Longitudinal evolution mean pairwise distance versus an external reference (HXB2R). Each boxplot shows results from HXB2 referenced pairwise distance for the four integrase amplicons. R/ape package, with a Kimura-80 model was used to calculate pairwise distances. Figure S2. Longitudinal evolution of summed Shannon Entropy values. Each boxplot shows results from Shannon Entropy values calculated and collapsed for the four integrase amplicons in a particular sample/timepoint combination. [file 1743-422X-10-350-S3.docx]

**Supplementary Figures**

**Figure S1.**Longitudinal evolution mean pairwise distance versus an external reference (HXB2R). Each boxplot shows results from HXB2 referenced pairwise distance for the four integrase amplicons. R/ape package, with a Kimura-80 model was used to calculate pairwise distances.

**Figure S2.** Longitudinal evolution of summed Shannon Entropy values. Each boxplot shows results from Shannon Entropy values calculated and collapsed for the four integrase amplicons in a particular sample/timepoint combination.

**Figure S1**


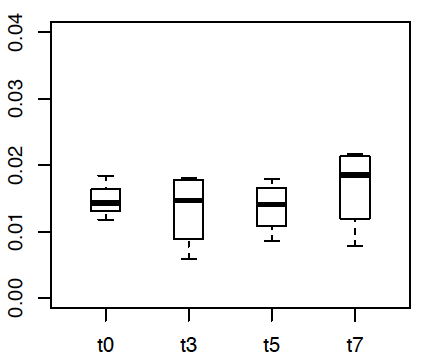

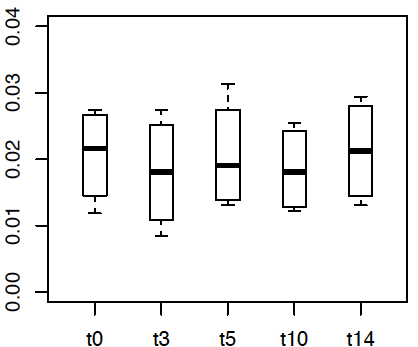

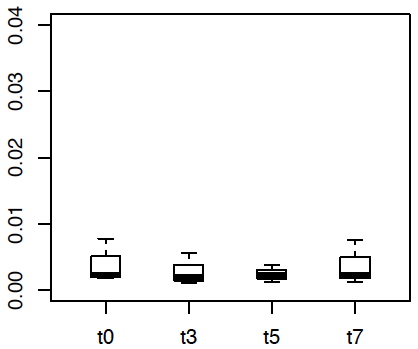

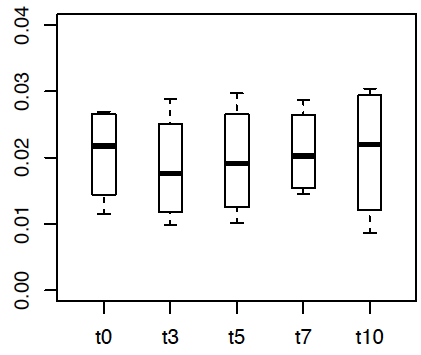

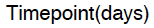

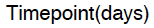

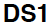

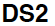

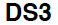

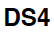


**0**

**3**

**5**

**7**

**0**

**3**

**5**

**10**

**14**

**0**

**3**

**5**

**7**

**0**

**3**

**5**

**7**

**10**

**Days after ART initiation**

**Mean Pairwise Distance**

**Mean Pairwise Distance**

**Figure S2**

| 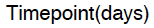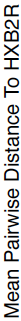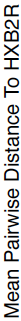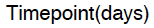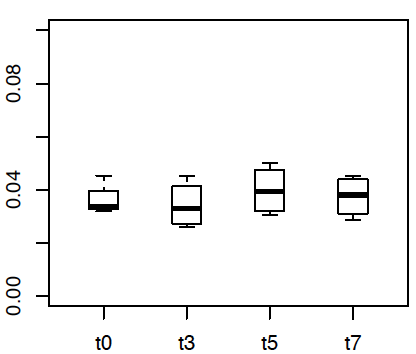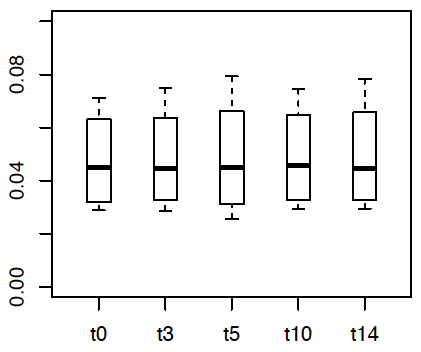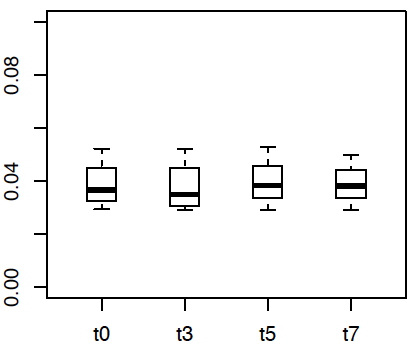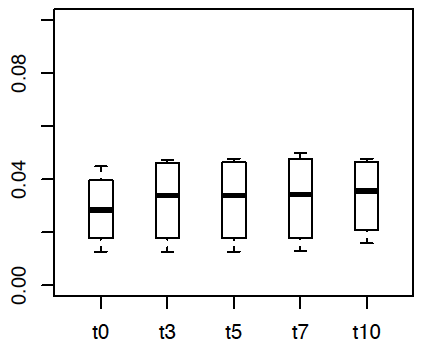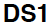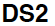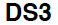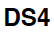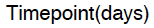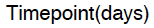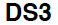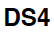 **0**  **3**  **5**  **7**  **0**  **3**  **5**  **10**  **14**  **0**  **3**  **5**  **7**  **0**  **3**  **5**  **7**  **10**  **Days after ART initiation** 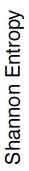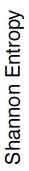 |
| --- |
